# La myosite ossifiante circonscrite du coude: à propos d’un cas

**DOI:** 10.11604/pamj.2016.24.320.5685

**Published:** 2016-08-19

**Authors:** Yassine Nhamoucha, Othmane Alaoui, Charifa Alaoui, Hicham Abdellaoui, Mohammed Tazi, Mohammed Oukhoya, Lamyae Chater, Karima Atarraf, Mounir Arroud, Abderahman Afifi

**Affiliations:** 1 Service de Chirurgie Pédiatrique 1, Hôpital Mère et Enfant, CHU de Fès, Maroc

**Keywords:** Myosite ossifiante, radiographie standard, coude, chirurgie, Myositis ossificans, standard radiography, elbow, surgery

## Abstract

La myosite ossifiante circonscrite (MOC) est une ossification hétérotopique des muscles striés. Sa localisation au niveau du coude est rare. Elle survient chez le sujet jeune, souvent suite à un traumatisme comme elle peut se développer également en dehors de tout traumatisme. Sa localisation prédominante est au niveau des muscles les plus volumineux de la racine des membres (fessiers, deltoïde) ou les plus exposés aux chocs direct (plus de 40 % des cas post-traumatiques sur le quadriceps). Nous proposons d’illustrer à partir d’une observation, les aspects que réalise la myosite ossifiante circonscrite en radiologie conventionnelle, en tomodensitométrie afin d’éviter la confusion diagnostique potentielle avec une tumeur osseuse maligne.

## Abstract (en)

Circumscribed myositis ossificans (CMO) is a heterotopic ossification of the striated muscles. Its location at the level of the elbow is rare. It occurs in young patients, often following trauma as it can also develop without experiencing any traumatic event. Its predominant location is at the level of the larger muscles limbs root (gluteus, deltoid) or of the areas which are most exposed to direct shocks (the quadriceps in more than 40% of post-traumatic cases). Our study aims to highlight the aspects of a circumscribed myositis ossificans in conventional radiology and tomodensitometry to avoid potential diagnostic confusion with a malignant bone tumor.

## Introduction

La myosite ossifiante circonscrite (MOC) est une ossification hétérotopique des muscles striés. C'est une affection bénigne pseudo-tumorale, rare, d'étiologie assez mal définie. C'est un processus local réactif, de développement auto limité, qui se produit à partir du tissu conjonctif interstitiel et non pas des fibres musculaire striées squelettiques. Le tableau clinique et la biologie ne sont pas spécifiques, ce qui rend à l'imagerie un rôle important dans le diagnostic de la maladie. Ce fut le cas de notre patient dont nous rapportons l'observation.

## Patient et observation

**Figure 1:**
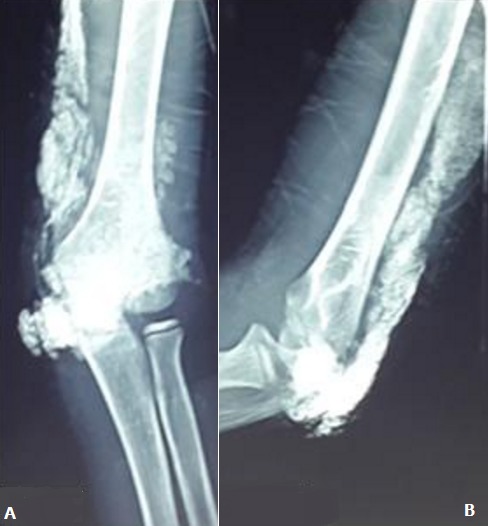
Cliché standard du coude de face (A) et de profil (B): montrant des calcifications en amas se projetant au niveau des parties molles de la face postérieure du coude sans lyse osseuse

**Figure 2:**
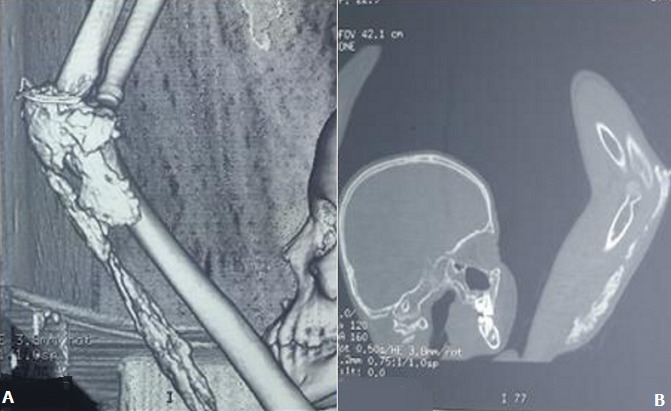
Coupes tomodensitométriques (A, B): formations de densité calcique en conglomérats intéressant la région postérieure du coude, respectant l’articulation radio-cubito-humérale

**Figure 3:**
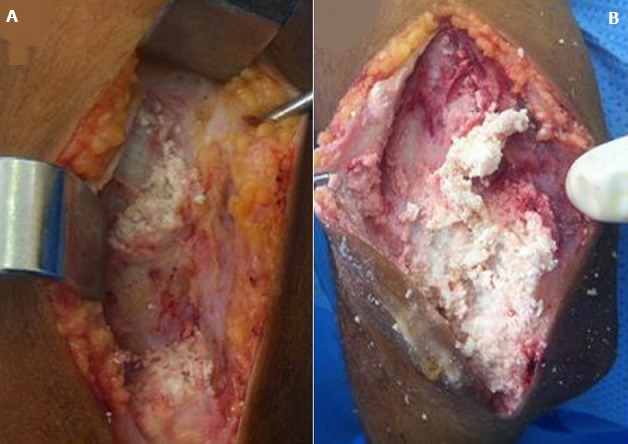
Image per-opératoire du coude (A et B): montrant des calcifications en amas se projetant au niveau sous cutané et au niveau de l’aponévrose du muscle triceps

**Figure 4:**
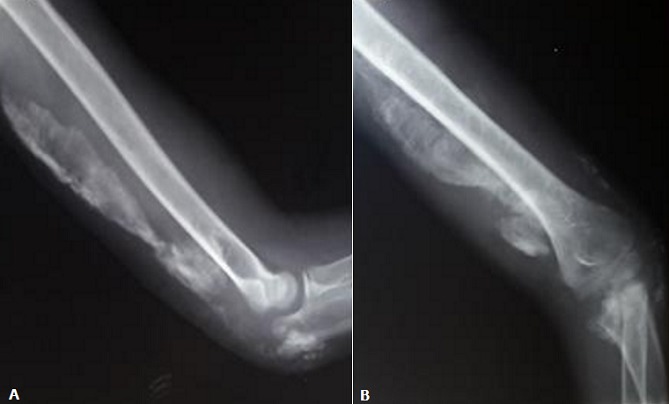
Cliché standard du coude de face (A) et de profil (B): montrant une diminution des calcifications en amas au niveau des parties molles de la face postérieure du coude

Il s'agit d'un enfant de 13 ans, connu suivi pour un syndrome néphrotique depuis l'âge de 8 ans avec bonne évolution, le début de sa symptomatologie remonte à 1 mois avant son admission ou l'enfant a été victime d'une chute de sa hauteur avec réception au niveau du coude occasionnant chez lui une impotence fonctionnelle partielle du membre supérieur gauche avec douleur et tuméfaction du coude ou il a été traité traditionnellement par un bandage et massage mais vu la non amélioration et l'aggravation de sa symptomatologie il a consulté au urgences pédiatriques de notre formation pour prise en charge. L'examen clinique à l'admission trouve un enfant conscient stable sur le plan hémodynamique et respiratoire avec un coude gauche tuméfié, douloureux, bloqué à 130°, avec des signes inflammatoires en regard. La radiographie du coude a montrée des calcifications périarticulaires du coude et au niveau de la partie postérieure du bras gauche sans fracture associée (Figure 1). Une échographie des parties molles a été réalisée et qui était sans particularité avec un bilan infectieux qui était ascensionné (CRP à 80 mg/l, globules blancs à 16 000 élément/mm^3^). Nous avons complété le bilan par une tomodensitométrie du coude qui a parlée de formations de densité calcique s'étend de la face postérieure du coude jusqu'au niveau de la partie moyenne du bras (Figure 2). L'exploration chirurgicale a constaté la présence de plusieurs calcification au niveau sous cutané et au niveau de l'aponévrose du muscle triceps (Figure 3) qu'on a reséqué. Les suites opératoires étaient simples et le contrôle radiologique post opératoire a montrée une diminution des calcifications (Figure 4). L'examen clinique après un mois a montré une légère amélioration des amplitudes de mobilité du coude avec un secteur de mobilité à environ 30°.

## Discussion

La myosite ossifiante circonscrite (MOC) est une pathologie bénigne, rare des parties molles, caractérisée par une prolifération hétérotopique non néoplasique d'os et de cartilage dans les tissus mous à distance du périoste. Cette affection est retrouvée avec une fréquence sensiblement égale dans les deux sexes, et concerne le sujet jeune jusqu´à 30 ans. La notion de traumatisme violent est parfois retrouvée mais souvent il s'agit de microtraumatismes répétés [1]. L'atteinte de la cuisse est la plus fréquente souvent dans le compartiment antérieur [2, 3]. L'atteinte du membre supérieur est rare [4]. Cliniquement la MOC se manifeste comme une masse douloureuse des parties molles, de survenue brutale, de volume d'emblée maximal accompagné de signes inflammatoires cliniques et biologiques. La douleur a tendance a décroitre avec l'évolution, ce qui fait la différence avec la pathologie tumorale [1]. L'aspect radiologique change parallèlement et est superposable à la maturation histologique de la lésion MOC. À la phase de début les radiographies sont normales, à la deuxième semaine apparaît une augmentation locale de densité et de volume des parties molles avec une possible réaction périostée de voisinage et de fines calcifications [5]. L'échographie peut montrer une masse ovalaire bien limitée hypoéchogène à centre échogène en rapport avec le phénomène de zone histologique. Le scanner est l'examen paraclinique de choix pour caractériser la minéralisation hétérotopique en mieux démontrant le phénomène de zone [6]. Dans les deux premières semaines la lésion apparaît comme une masse relativement hypodense sans calcification centrale ou périphérique. À ce stade l''dème périlésionnel peut être vu mais il est mieux apprécié par l'IRM. La disposition en anneau périphérique des calcifications est plus précocement et plus facilement mise en évidence que sur les clichés simples. L'os sous-jacent n'est pas envahi mais peut présenter une encoche ou une réaction périostée en regard [7]. Le diagnostic différentiel chez l'enfant devant présentant des images calciques douloureuses proches de la diaphyse des os longs, il conviendra d'éliminer une tumeur maligne [7, 8]. La MOC est d'évolution spontanément favorable. L'exérèse chirurgicale des ostéomes n'est pas systématique. La chirurgie est indiquée en cas de compression neurologique ou de raideur articulaire. Dans ce cas, la scintigraphie est indispensable pour vérifier la maturation des lésions [6].

## Conclusion

La MOC est une affection bénigne des parties molles, dont l'étiologie et dont l'évolution est constamment favorable. Le tableau clinique et paraclinique initiale de la MOC fait craindre un processus malin ce qui peut amener parfois a une biopsie chirurgicale. Le traitement reste essentiellement médical, il est rarement chirurgical.
